# A Case of Pseudomonas mendocina Bacteremia in an Elderly Man With Bilateral Leg Lesions

**DOI:** 10.7759/cureus.17777

**Published:** 2021-09-06

**Authors:** Ekene U Ezeokoli, Mustafa U Polat, Olusola Ogundipe, John Szela

**Affiliations:** 1 Infectious Disease, Oakland University William Beaumont School of Medicine, Royal Oak, USA; 2 Infectious Disease, Beaumont Hospital, Royal Oak, USA

**Keywords:** pseudomonas, mendocina, psuedomonas mendocina, bacteremia, sepsis, ulcers, soft tissue

## Abstract

*Pseudomonas mendocina* is a gram-negative, aerobic, rod-shaped bacterium that rarely causes disease in humans. Documented infections can be severe with varying etiologies, often requiring intensive care. We describe a rare case of bacteremia with *P. mendocina *in an elderly male, with a comprehensive review of the literature.

An 81-year-old Caucasian male presented with bilateral lower leg erythema and drainage but was afebrile. His past medical history included atrial fibrillation, chronic kidney disease, and congestive heart failure. Labs showed leukocytosis and a blood culture was obtained revealing *Pseudomonas mendocina*. The pathogen was susceptible to all antibiotics tested and he was successfully treated on cefepime inpatient and a two-week course of ciprofloxacin on discharge.

Our case and literature review presents a successful treatment of a rare cause of bacteremia likely stemming from a soft tissue nidus. *P. mendocina* has a favorable susceptibility profile and the antibiotics preferred differ from *Pseudomonas aeruginosa*, a more common pathogen. Worldwide there have been only 18 other documented cases of *P. mendocina* infection, all successful and with no mortality. Physicians can confidently utilize usual antibiotics in the treatment of this pathogen despite its rare clinical manifestations.

## Introduction

*Pseudomonas mendocina* is a gram-negative, aerobic, rod-shaped bacterium. It is found naturally in water and soil, with a range of survivability in environments and was first classified in Argentina in 1970 [1]. Human infection is rare, but can be severe and with associated morbidity and mortality. It is ubiquitous in the environment with documented disease in Europe [2], North America [3-5] and Asia [6,7].

Fortunately, *P. mendocina* has a favorable resistance profile [8]. This is likely at least in part from the absence of selective antimicrobial pressure because of the rarity of human infection. As of the time of literature review, there have only been a handful of documented cases of human *P. mendocina* infection in the United States. We describe the clinical course of a case of *P. mendocina* in an adult at a tertiary health care facility.

## Case presentation

An 81-year-old male presented from an extended care facility to the emergency department of our institution with bilateral lower extremity pain, erythema, and swelling. The patient had an extensive medical history including coronary artery disease, atrial fibrillation, systolic and diastolic heart failure with an ejection fraction of 35%, stage 3 chronic kidney disease, diabetes mellitus type 2, and previous strokes. He reported one week of rapid evolution of longstanding lower extremity edema with the development of blisters with oozing sanguineous fluid. He was unaware of any fever episodes but reported chills, nausea, and vomiting whenever he tried to eat. He did report trauma to his lower limbs from falls and had received inconsistent care of his wounds.

At the time of presentation, the patient was hemodynamically stable but with a mild tachycardia with a heart rate of 103. Other vitals were: temperature 97.6° F, blood pressure of 111/59, respiratory rate of 23 with an oxygen saturation of 100% on room air. On physical exam, he appeared nontoxic, alert, and awake. His breathing was non-labored and lungs were clear to auscultation. His cardiac exam, with the exception of tachycardia, was benign, without heave, thrill, gallop or murmur. His left lower extremity was erythematous from the knee and distally, along with multiple bullae and areas of skin desquamation with sanguineous drainage. His right lower limb was also somewhat erythematous but much less so as compared to the left. There was notable tenderness to palpation of both extremities.

Laboratory evaluation revealed leukocytosis with a WBC count of 24,500/mm^3^, acute kidney injury complicating his pre-existing chronic kidney disease as well as a mild lactic acidemia of 3.3. He was diagnosed with sepsis secondary to cellulitis and was admitted. Imaging studies were performed including bilateral venous Doppler which was negative for deep vein thrombosis and superficial thrombophlebitis. Radiographs of the left tibia and fibula showed no fracture, dislocation or bony destruction, but noted diffuse soft tissue swelling. He was started on intravenous vancomycin as dosed by our pharmacy. 2/2 pre-antibiotic blood cultures collected on admission subsequently grew *Pseudomonas mendocina*. An antibiotic susceptibility profile was performed and is summarized in Table 1. Infectious diseases teaching service was consulted, intravenous vancomycin was stopped, and the patient was started on cefepime 2g every 12 hours. Blood cultures were repeated 48 hours after initiation of treatment and were negative. At 72 hours of admission and antibiotic therapy, the patient had significant improvement of leukocytosis with a WBC count down to 15,100/mm^3^. The patient also received intravenous furosemide for management of heart failure symptoms with improvement of lower limb edema. He was discharged in stable condition to a subacute rehab facility with instructions to complete two weeks of antibiotics therapy with oral ciprofloxacin.

**Table 1:** Pseudomonas mendocina antibiotic susceptibility in our patient MIC: Minimum inhibitory concentration

Antibiotic	MIC (ug/ml)	Interpretation
Cefepime	<=2	Susceptible
Ciprofloxacin	<=1	Susceptible
Gentamicin	<=1	Susceptible
Levofloxacin	<=0.25	Susceptible
Piperacillin/Tazobactam	<=4	Susceptible
Tetracycline	<=4	Susceptible
Tobramycin	<=1	Susceptible

## Discussion

*Pseudomonas mendocina* is an uncommon pathogen and is a very rare cause of infection in humans. Our review of literature returned 18 reported cases of *P. mendocina* in humans (Table 2) [8].

**Table 2:** Literature on P. mendocina

Publication Year	Location	Age/Sex	Infection type	Treatment regimen
1992 [9]	Argentina	63/Male	Endocarditis	IV ceftriaxone + IV gentamicin x6 weeks followed by oral ciprofloxacin x2 weeks
2001 [2]	Denmark	28/Female	Endocarditis	IV gentamicin + IV ampicillin followed by ciprofloxacin
2005 [10]	Taiwan	65/Male	Spondylodiscitis	IV cefepime x6 weeks, followed by oral ciprofloxacin x4 weeks
2007 [11]	Turkey	36/Male	Endocarditis	IV ceftazidime + IV amikacin x6 weeks
2011 [12]	France	79/Female	Endocarditis	IV piperacillin + IV gentamicin x6 weeks
2011 [13]	Israel	31/Male	Bacteremia	IV gentamicin + oral ofloxacin x2 weeks
2012 [6]	Singapore	86/Female	Osteomyelitis	N/A
2013 [7]	Singapore	34/Male	Septic arthritis	Oral trimethoprim sulfamethoxazole x16 days
2016 [3]	United States	57/Male	Endocarditis	IV piperacillin/tazobactam x6 weeks
2017 [14]	Portugal	22/Male	Peritonitis	Intraperitoneal cefazolin + intraperitoneal ceftazidime followed by oral ciprofloxacin x3 weeks
2018 [15]	Argentina	56/Male	Skin/soft tissue	Ampicillin sulbactam/colistin
2018 [15]	Argentina	36/Male	Skin/soft tissue	Colistin
2018 [16]	Taiwan	55/Male	Meningitis	IV ceftriaxone
2018 [16]	Taiwan	66/Female	Meningitis	IV ceftazidime
2018 [16]	Taiwan	79/Male	Meningitis	IV meropenem
2018 [16]	Taiwan	78/Female	Meningitis	IV cefepime
2019 [4]	United States	63/Male	Bacteremia	Ceftazidime x10 days
2020 [5]	United States	72/Male	Bacteremia	IV piperacillin/tazobactam followed by oral ciprofloxacin x2 weeks
This report	United States	81/Male	Bacteremia	IV cefepime followed by oral ciprofloxacin x2 weeks

Endocarditis (28%), meningitis (22%), and bacteremia (22%) appear to be the most common clinical presentations, but a wide variety of infection sites are possible as noted in Table 2. A systematic review in 2020 by Ioannou and Vougiouklakis [8] describes the literature, with no known cases of death, and the very rare resistance to usual antibiotics.

Our patient presented with bacteremia with a suspected source of infection believed to be his skin lesions with subsequent hematogenous spread. This case adds to the literature of rare human infection with an uncommon organism that was successfully treated and will help advise future clinical approaches to *P. mendocina* infection in humans.

## Conclusions

*P. mendocina* is an uncommon Gram-negative bacterium implicated in bacteremia, cardiac, central nervous system, skin, and soft tissue infections. Our patient presented with bacteremia likely from hematogenous spread from his lower extremity skin wounds. He was successfully treated and had an uncomplicated clinical course. *P. mendocina* shows sensitivity to most antibiotics except ampicillin. Physicians can confidently utilize usual antibiotics in the treatment of this pathogen despite its rare clinical manifestations.
